# The interaction between Carbohydrates and the Antimicrobial Peptide P-113Tri is Involved in the Killing of *Candida albicans*

**DOI:** 10.3390/microorganisms8020299

**Published:** 2020-02-21

**Authors:** Guan-Yu Lin, Chuan-Fa Chang, Chung-Yu Lan

**Affiliations:** 1 Institute of Molecular and Cellular Biology, National Tsing Hua University, Hsinchu 30013, Taiwan; legendsun3786@gmail.com; 2 Department of Medical Laboratory Science and Biotechnology, National Cheng Kung University, Tainan 70101, Taiwan; affa@mail.ncku.edu.tw; 3 Department of Life Science, National Tsing Hua University, Hsinchu 30013, Taiwan

**Keywords:** *Candida albicans*, antimicrobial peptide, P-113Tri, cell wall, mannan

## Abstract

The emergence of drug resistance to *Candida albicans* is problematic in the clinical setting. Therefore, developing new antifungal drugs is in high demand. Our previous work indicated that the antimicrobial peptide P-113Tri exhibited higher antifungal activity against planktonic cells, biofilm cells, and clinical isolates of *Candida* species compared to its parental peptide P-113. In this study, we further investigated the difference between these two peptides in their mechanisms against *C. albicans*. Microscopic examination showed that P-113 rapidly gained access to *C. albicans* cells. However, most of the P-113Tri remained on the cell surface. Moreover, using a range of cell wall-defective mutants and competition assays, the results indicated that phosphomannan and N-linked mannan in the cell wall are important for peptide binding to *C. albicans* cells. Furthermore, the addition of exogenous phosphosugars reduced the efficacy of the peptide, suggesting that negatively charged phosphosugars also contributed to the peptide binding to the cell wall polysaccharides. Finally, using a glycan array, P-113Tri, but not P-113, can bind to other glycans commonly present on other microbial and mammalian cells. Together, these results suggest that P-113 and P-113Tri have fundamental differences in their interaction with *C. albicans* and candidacidal activities.

## 1. Introduction

*Candida* species are associated with a range of clinical manifestations, including mucosal and invasive bloodstream infections [1]. Among the *Candida* species, *Candida albicans* is a leading cause of bloodstream infections, although the incidence of infections caused by non-*albicans Candida* species is increasing [1]. Moreover, because there are limited drug classes available for the treatment of *Candida* infections, and due to the overuse of antifungals, the emergence of drug resistance is becoming a significant concern in clinical settings [2,3]. Antimicrobial peptides (AMPs) have been identified in virtually all organisms and have diverse structures and functions, such as antimicrobial and immunomodulatory activities [4,5,6,7,8]. Because AMPs exhibit broad-spectrum activity against microorganisms and insusceptibility to conventional drug resistance mechanisms, AMPs are promising candidates for the development of new antifungal drugs [9,10,11,12,13].

Human histatin 5 (Hst 5) is a naturally occurring protein found in human saliva that exhibits potent antifungal activity. P-113, a peptide containing 12 Hst 5 amino acid residues, retains full candidacidal activity and has had no adverse effects in clinical trials [14,15]. However, the efficacy of P-113 is significantly reduced in the presence of high salt concentrations and at pH 4.5 [16,17,18]. In our previous study, novel P-113 derivatives, such as P-113Tri (a tandem arrangement of three P-113 repeats) were synthesized and characterized [19]. P-113Tri contained significant fractions of an α–helical conformation and was more resistant to high salt and low pH than P-113 [19] and Figure S1. Moreover, compared to P-113, P-113Tri exhibited increased antifungal activity against planktonic cells, biofilm cells, and clinical isolates of *C. albicans* and non-*albicans Candida* species [19]. However, the detailed mechanism by which P-113Tri functions differently from P-113 in its anti-*C. albicans* activity is still unknown. In this work, we aim to study the difference between P-113Tri and P-113. We showed that P-113 rapidly gains access to the cells where it accumulates. However, although small amounts of P-113Tri slowly gained access to the cells, most of the P-113Tri remained associated with the *C. albicans* cell surface. Particularly, P-113Tri interacted with the glycan components of the cell wall. In addition, the interaction between P-113Tri and the cell wall carbohydrates was somehow correlated with the candidacidal activity of P-113Tri. These results enhance our understanding of how an AMP attacks *C. albicans* through its interaction with the glycans present in fungal pathogens. Moreover, our findings suggest the potential use of P-113Tri as a new therapeutic agent that can target the cell wall carbohydrates of fungal pathogens.

## 2. Materials and Methods

### 2.1. Antifungal Peptides and Reagents

P-113, P-113Tri, fluorescein isothiocyanate (FITC)-P-113, and FITC-P-113Tri were synthesized by Mission Biotech System (Taipei, Taiwan). FITC is conjugated to the N-terminus of the peptides. The purities of these peptides were analyzed by reversed-phase high-performance liquid chromatography and mass spectrometry to be >95% pure. All reagents were obtained from Sigma-Aldrich unless indicated otherwise.

### 2.2. C. albicans Strains and Growth Media

All *C. albicans* strains used in this study are listed in Table S1. Cells were routinely grown in YPD medium (2% glucose, 1% yeast extract, and 2% peptone). Plates were prepared with 1.5% agar. For the minimum inhibitory concentration (MIC) assay, LYM broth (5.4 mM KCl, 5.6 mM Na_2_HPO_4_, 0.5 mM magnesium sulfate, 1.0 mM sodium citrate, 0.4 mg of ZnCl_2_, 2.0 mg of FeCl_3_·6H_2_O, 0.1 mg of CuSO_4_·5H_2_O, 0.1 mg of MnSO_4_·H_2_O, and 0.1 mg of Na_2_B_4_O_7_·10H_2_O, 2% glucose, amino acid mixture and a vitamin mixture, all per liter of medium) was used [14]. The amino acid mixture and vitamin mixture were purchased from Thermo Fisher Scientific (Waltham, MA, USA).

### 2.3. C. albicans Killing Assay

The killing assays were performed as previously described [19]. Briefly, *C. albicans* cells were grown overnight in YPD medium at 30 °C with shaking, subcultured into fresh YPD and further grown to the exponential phase (~5 h). Then, the cells were treated with or without AMPs for 1 h. The number of viable cells after peptide treatment were normalized to those of control cells (no peptide treatment) and are reported as percentages.

### 2.4. Measurement of Minimum Inhibitory Concentrations (MICs)

The MICs for each peptide were determined using the Clinical and Laboratory Standards Institute (CLSI) method M27-A3 [20] with some modifications. Cells were inoculated into each well of 96-well plates in LYM broth (4 × 10^4^ cells/mL) with or without different concentrations of peptides. The cells were grown at 37 °C for 48 h and examined by visual estimation and spectrophotometric determination. The MIC_90_ values of the AMPs were defined as the concentrations of the peptides that caused a 90% reduction in cell growth relative to the control cells without peptide treatment. The assays were performed independently at least three times for each experimental group.

### 2.5. Confocal Scanning Laser Microscopy

To examine the cellular localization of the peptides, *C. albicans* cells were grown to the exponential phase, harvested by centrifugation, and resuspended in 12.5 mM sodium acetate to reach a concentration of 2.4 × 10^8^ cells/mL. Then, cells were added to 25 μM CellTracker Blue 7-amino-4-chloromethylcoumarin (CMAC; Invitrogen) or 8 μg/mL calcofluor white (CFW; Sigma-Aldrich F-3543) and further grown at 30 °C for 30 min in the dark. CellTracker Blue CMAC and CFW were used to stain yeast vacuoles [21] and cell wall chitin, respectively. After centrifugation (12,000× *g*, 5 min) to remove the unbound dyes, cell pellets were collected, washed three times with 12.5 mM sodium acetate, and resuspended in 12.5 mM sodium acetate. Finally, FITC-conjugated peptides were added to the cells, as indicated. The colocalization of FITC-conjugated peptides to the vacuoles or cell walls was examined using a Zeiss LSM 800 Airyscan confocal microscope. The images were processed and analyzed using Zen software (Zeiss).

### 2.6. Binding of P-113 and P-113Tri to C. albicans Cells

The binding of AMPs to *C. albicans* was determined as previously described [22]. Briefly, *C. albicans* (6 × 10^7^ cells) were incubated with Zymolyase-20T (2.5 mg/mL) in SCE buffer (1 M Sorbitol, 10 mM sodium citrate buffer [pH 6.0] and 1 mM EDTA) at 37 °C for 1 h, followed by washing twice with SCE buffer. To remove the carbohydrates from the cell wall, cells were treated with concanavalin A (100 μg/mL in 12.5 mM sodium acetate) at 30 °C for 1 h and then washed twice with 12.5 mM sodium acetate. For metaperiodate treatment, cells were suspended in 50 mM sodium acetate (pH 4.5) containing 100 mM metaperiodate and incubated at 4 °C for 30 min in the dark and then washed twice with 12.5 mM sodium acetate. For α1-2,3,6 mannosidase treatment, cells were inoculated in GlycoBuffer 4 (New England Biolabs, Madison, WI) containing 5 U of α-mannosidase, incubated at 37 °C for 16 h, and washed twice with 12.5 mM sodium acetate. Then, *C. albicans* cells (2.4 × 10^8^ cells/mL) were treated with FITC-P-113 and FITC-P-113Tri (0.6 μg/mL in 12.5 mM sodium acetate) for 2 min. Subsequently, an Accuri C6 flow cytometer (BD Biosciences) was used to determine the extent of the peptides bound to *C. albicans* cells by calculating the mean fluorescence intensity (MFI) from 10,000 cells per sample. The relative peptide binding to the cells is presented as a percentage by dividing the MFI of the cells treated with peptide by that of the control without peptide treatment. Finally, the cells from the peptide binding assay described above were also treated with 20 μg/mL propidium iodide (PI) to determine the extent of membrane disruption upon peptide treatment, as previously described [23].

### 2.7. β-Glucan Staining

To stain for total β-Glucan, cells were incubated with aniline blue fluorochrome (Wako; 500 μg/mL) for 5 min in a black 96-well microplate. Aniline blue fluorescence intensity was measured using a VICTOR3 Multilabel Plate Reader fluorescence spectrophotometer (PerkinElmer) with excitation and emission wavelengths of 405 nm and 460 nm, respectively.

### 2.8. Competition Assays

For the competition assays, P-113 and P-113Tri (12 μg/mL) were preincubated with 1, 2, 4, and 8 mg/mL mannan and laminarin at 4 °C for 30 min, followed by mixing with cells and incubating at 37 °C for 1 h. Then, the numbers of viable cells after peptide treatment were normalized to those of the control cells (no peptide treatment) and are reported as percentages. All carbohydrate stock solutions were prepared in 12.5 mM sodium acetate. Mannan from *Saccharomyces cerevisiae* and Laminarin from Laminaria digitata were purchased from Sigma-Aldrich (catalog numbers M7504 and L9634).

### 2.9. Measurement of the Dissociation Constants for the Peptide/Glycan Complexes

Dissociation constants (K_D_) for the peptide/glycan complexes were measured using isothermal titration calorimetry (ITC). Thermodynamic analysis of peptide binding to mannan and laminarin was performed using a MicroCal iTC200 calorimeter (MicroCal, Northampton, MA) as previously described [24]. All experiments were performed at 25 °C using P-113 (25 μM), P-113Tri (25 μM), mannan (116 μM) and laminarin (130 μM). The P-113 or P-113Tri solution was placed in a calorimeter cell, and the mannan or laminarin solution was loaded into the syringe injector. In an individual titration, the autocontrolled microsyringe injected 2 μL of mannan or laminarin solution into the peptide solution over an interval of 120 s. The integrated heat change was analyzed by means of nonlinear regression using MicroCal Origin software, and the dissociation constant (K_D_) was obtained from a single sigmoidal titration curve. The effect of the dilution of the mannan or laminarin solution in the titration cell was removed by subtracting the calorimetric data for a blank titration, which consisted of the titration of the mannan or laminarin solution into sodium acetate.

### 2.10. Glycan Microarray Analysis

Glycan array screening was carried out using a rapid, nonwashing, solution carbohydrate array as previously described [25]. Briefly, donor beads (500 ng/well) and biotin-polyacrylamide (PAA)-sugars (20 ng/well) (GlycoTech, Gaithersburg, MD, USA) mixed with FITC-P-113Tri (40 ng/well) were incubated for 1 h (a total of 15 μL of reaction solution). A mixture of acceptor beads (500 ng/well), mouse anti-FITC antibody (50 ng/well) and rabbit anti-mouse IgG antibody (25 ng/well) (Zymed, San Francisco, CA) was added to the reaction to reach a final volume of 25 μL. All reactions were performed in the dark. After incubation for 2 h, the peptide binding signals were measured from a PerkinElmer EnVision instrument and analyzed using the AlphaScreenTM detection program. The results are expressed as the fluorescence intensities.

### 2.11. Statistical Analysis

Data were assessed for statistical significance by the two-tailed Student’s *t*-test.

## 3. Results

### 3.1. P-113Tri Directly Interacts with the Cell Surface of C. albicans

**Figure 1:**
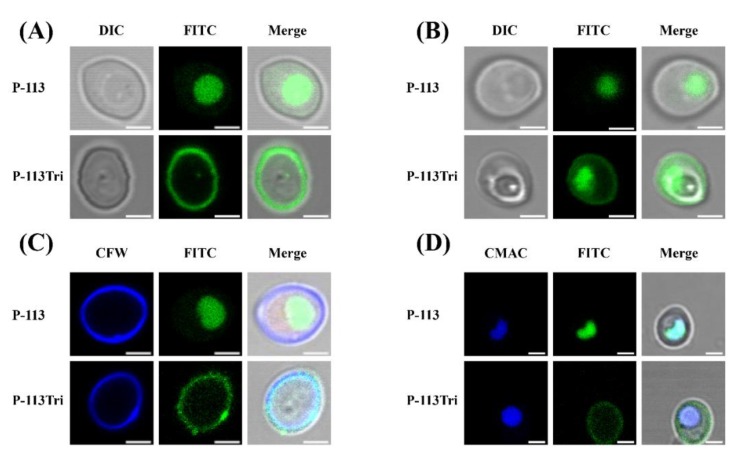
Examination of the interactions of P-113 and P-113Tri with *Candida albicans* cells using confocal microscopy. (**A**) Differential interference contrast (DIC) and fluorescence images show that FITC-P-113 quickly gains entry into the cells within 5 min. (**B**) FITC-P-113 and a part of FITC-P-113Tri accumulated in vacuoles after 1 h of treatment. (**C**) FITC-P-113Tri was found to bind to the cell wall, as demonstrated by colocalization with calcofluor white. (**D**) The peptides inside the cells accumulated in vacuoles as demonstrated by colocalization with CMAC. Scale bar, 2 μm.

In our previous study, P-113Tri showed overall higher anti-candidacidal activity than its parental compound P-113 [19]. To further investigate the mechanisms of P-113Tri against *C*. *albicans*, the interaction between the cells and peptides conjugated with fluorescein isothiocyanate (FITC) was determined using confocal microscopy. After cell incubation with peptides for approximately 5 min, P-113 readily gained access into the cells, whereas P-113Tri remained on the cell surface (Figure 1A). However, after incubation for approximately 1 h, although small amounts of P-113Tri were present intracellularly, the majority of P-113Tri was still retained on the cell surface (Figure 1B). To verify this finding, the cellular localization of FITC-conjugated peptides was determined by cell staining with calcofluor white (CFW) and CellTracker Blue CMAC. CFW and CellTracker Blue CMAC are fluorescent dyes that selectively stain the cell wall and the lumen of yeast vacuoles, respectively. Figure 1C,D show that P-113Tri mainly interacted with the cell wall, although small amounts of P-113Tri gained intracellular entry, as exemplified by its colocalization with vacuoles. These results suggest that P-113Tri binds to the outer surface of *Candida* cells.

### 3.2. P-113Tri Binding to C. albicans Cell Wall Carbohydrates Is Related to the Candidacidal Activity of the Peptide

The cell wall is the outermost layer of *C. albicans* and plays a key role in interacting with the environment and host cells. Carbohydrates are the major components of the *C. albicans* cell wall, comprising 80% to 90% of the wall, and contain mannans, β-1,6-glucan, β-1,3-glucan, and chitin. Mannan is commonly the glycoprotein carbohydrate portion of the cell wall found with the following three structures: Linear *O*-linked mannan, highly branched *N*-linked mannan, and phosphomannan [26]. The glucan and chitin layers of the *C. albicans* cell wall are buried beneath a thin but electron dense mannan layer [27].

**Figure 2:**
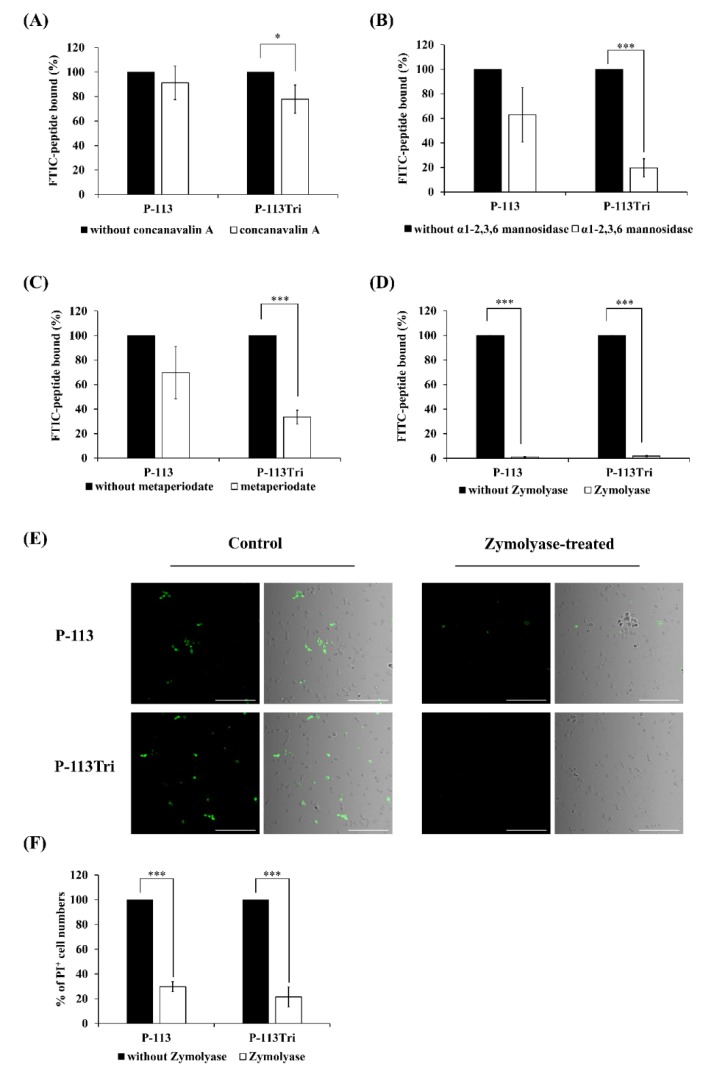
Effect of *C*. *albicans* cell wall modifications on the peptides cell binding and antifungal activity. (**A**) The binding of FITC-P-113 and FITC-P-113Tri to concanavalin A-treated *C. albicans* cells. *C. albicans* were treated with concanavalin A (100 μg/mL) at 30 °C for 1 h and then incubated with the peptides (0.6 μg/mL). (**B**) The binding of FITC-P-113 and FITC-P-113Tri to α1-2,3,6 mannosidase-treated *C. albicans*. Cells were treated with α1-2,3,6 mannosidase (5.0 U) at 37 °C for 16 h and then incubated with the peptides (0.6 μg/mL). (**C**) The binding of FITC-P-113 and FITC-P-113Tri to metaperiodate-treated *C. albicans*. Cells were treated with metaperiodate (100 mM) for 30 min at 4 °C and then incubated with the peptides (0.6 μg/mL). (**D**) The binding of FITC-P-113 and FITC-P-113Tri to Zymolyase-treated *C. albicans*. Cells were treated with Zymolyase (2.5 mg/mL) for 1 h at 37 °C and then incubated with the peptides (0.6 μg/mL). (**E**) Confocal microscopic examination of the interaction of P-113 and P-113Tri with Zymolyase-treated *C. albicans*. Cells were treated with Zymolyase (2.5 mg/mL) for 1 h at 37 °C and incubated with the peptides (0.6 μg/mL). Scale bar, 50 μm. (**F**) Viability of Zymolyase-treated cells with peptide treatment. *C. albicans* were treated with Zymolyase (2.5 mg/mL) for 1 h at 37 °C and incubated with 20 μg/mL propidium iodide (PI) and peptides (0.6 μg/mL).

Because the peptides colocalized with the cell wall (Figure 1A−D), it raises the possibility that P-113Tri interacts with components of the cell wall. To test this hypothesis, different parts of the cell wall carbohydrates were removed. Concanavalin A (ConA) specifically binds to the α-D-glucose and α-D-mannose residues of cell wall glycoproteins, and α1-2,3,6 mannosidase is an exoglycosidase that can degrade the mannan network. As shown in Figure 2A, the amount of FITC-P-113Tri bound to the ConA-treated *C*. *albicans* cells was significantly reduced compared to that in the controls without ConA treatment or with P-113 treatment. Moreover, the FITC-conjugated P-113 and P-113Tri bound to cells treated with α1-2,3,6 mannosidase were decreased by 40% and 80%, respectively, compared to the untreated controls (Figure 2B). These results confirm the interaction between the peptides we tested and mannan in the *Candida* cell wall.

To further examine the peptides and cell wall carbohydrate interactions, Zymolyase and sodium metaperiodate were also used. Zymolyase (or laminaripentaohydrolase) can hydrolyze the β-1,3-glucan layer, and sodium metaperiodate oxidizes cleaves the glycan chains of β-1,6-glucan and mannan. The extent of cell wall disruption by Zymolyase was assessed by aniline blue staining [28]. After deglycosylation by Zymolyase, the amount of cell-surface-bound aniline blue decreased by ~50%, representing the removal of ~50% of the cell wall glucan (Figure S2). Interestingly, the binding of FITC-conjugated P-113 and P-113Tri to the metaperiodate-treated cells decreased by 30% and 70%, respectively (Figure 2C). Similarly, the fluorescence intensity of FITC-conjugated peptides detected by a confocal microscope indicated that the binding of both FITC-conjugated peptides P-113 and P-113Tri to the Zymolyase-treated cells was significantly reduced compared to their binding to cells without peptide treatment (Figure 2D,E). In addition, cells treated with Zymolyase were more resistant to the peptides compared to the untreated cells, as determined by PI staining (Figure 2F).

**Figure 3:**
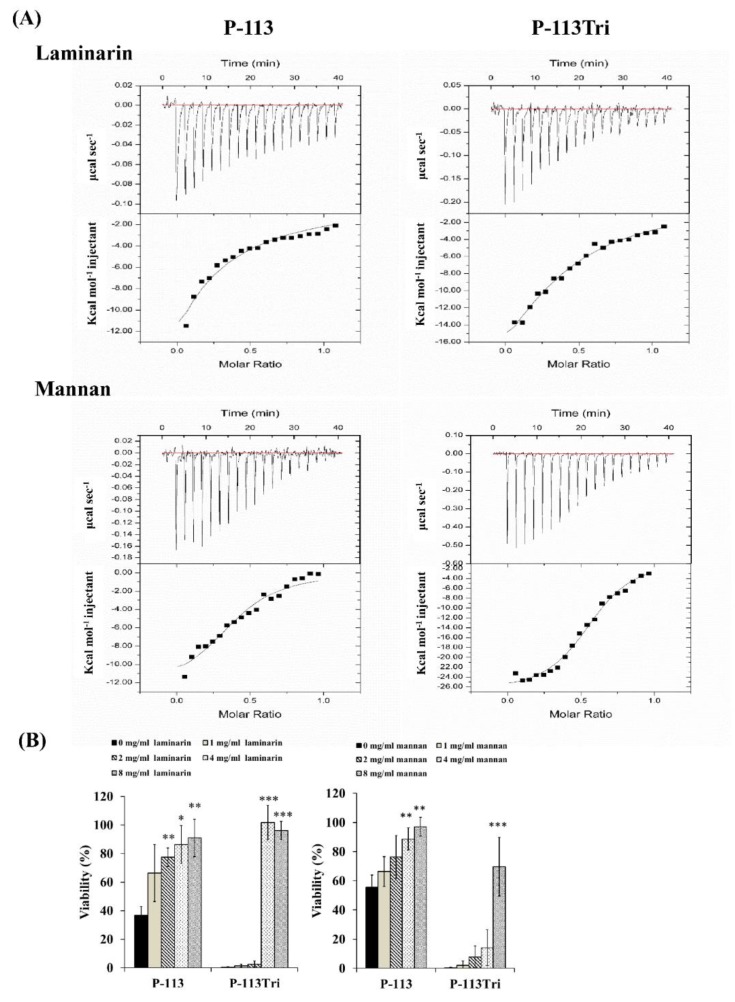
P-113 and P-113Tri bind to mannan and laminarin. (**A**) The interaction of the peptides with mannan and laminarin was measured by isothermal titration calorimetry (ITC). (**B**) Viability of *C*. *albicans* cells treated with different concentrations of mannan and laminarin and the peptides (12 μg/mL).

The dissociation constant (K_D_) for the carbohydrate/peptide complexes was further determined using isothermal titration calorimetry (ITC). The K_D_ values for the complexes of laminarin/P-113 and laminarin/P-113Tri were 33.2 μM and 11.33 μM, respectively (Figure 3A). Laminarin is a polysaccharide of glucose and a representative of β-1,3-glucan. The K_D_ values for the complex of mannan/P-113 and mannan/P-113Tri were 2.51 μM and 1.07 μM, respectively. Moreover, the K_D_ values of the laminarin/peptide complex are much higher than those for the mannan/peptide, suggesting that the peptides preferentially bind to *C. albicans* mannan rather than glucan.

Finally, to correlate the specific peptide–carbohydrate binding to the candidacidal activity, a competition assay was performed. Mannan and laminarin were premixed with the peptides at 4 °C for 30 min, followed by incubation of the peptide/carbohydrate complex with *Candida* cells. As shown in Figure 3B, although the laminarin and mannan concentrations varied, these polysaccharides rescued the cells from the candidacidal effect of the peptides. Taken together, our results indicate that the peptides interact with mannan and β-1,3-glucan of the cell wall, and this carbohydrate–peptide binding is related to the anti-*Candida* activity of the tested peptides.

### 3.3. Peptides Bind to N-Linked Mannan But Not O-Linked Mannan and Play a Partial Role in Candidacidal Activity

**Figure 4:**
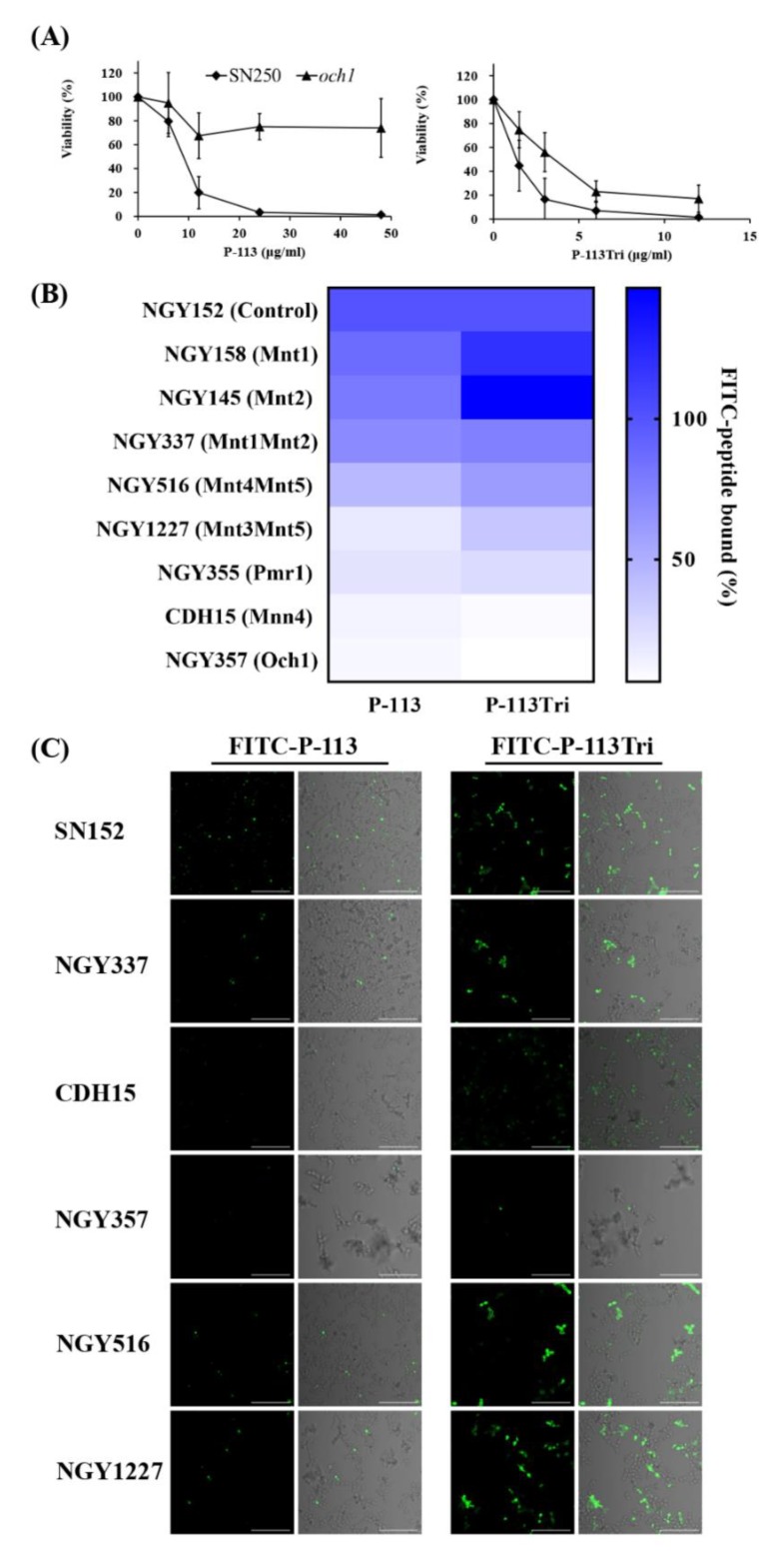
Effect of cell wall mannan modification on the peptides and their cell binding and antifungal activity. (**A**) Viability of the *C. albicans* *och1*-deletion mutant after treatment with P-113 and P-113Tri. Cells were treated with different concentrations of the peptides at 37 °C for 1 h. (**B**) The binding of FITC-P-113 and FITC-P-113Tri to various glycosylation mutants. Cells were incubated with FITC-labeled peptides (0.6 μg/mL). (**C**) Confocal microscopic examination to demonstrate the interaction of FITC-P-113 and FITC-P-113Tri with *C*. *albicans* glycosylation mutants. Cells were incubated with FITC-peptides (0.6 μg/mL). Scale bar, 50 μm.

To identify potential cell wall targets of the peptides, we prescreened 560 strains from *C. albicans* deletion mutant libraries [29,30]. *C*. *albicans* cells were incubated in 12.5 mM sodium acetate with or without P-113 (16 μM) for 1 h. The *C*. *albicans* wild type strains (SC5314 and SN250) were sensitive to P-113, whereas 15 deletion mutants were resistant to P-113. Among these resistant strains, many of them lack genes encoding cell wall proteins (Table 1), for example, the *och1*-deletion mutant. The *OCH1* gene encodes an α-1,6-mannosyltransferase that initiates elongation of the *N*-linked mannan outer chain of the *C*. *albicans* cell wall [26]. Figure 4A shows that the *och1*-deletion mutant was more resistant to P-113 compared to the wild-type SN250 strain. Interestingly, the candidacidal activity of P-113Tri to the *och1*-deletion mutant seems to be even better than P-113 (Figure 4A). These results further suggest that the mannosylation of cell wall proteins is related to the activity of the peptides.

To further reveal the relationship between mannosylation-defective mutants and FITC-conjugated peptides, binding assays were performed by flow cytometry. The *C*. *albicans* cells were treated with 0.6 μg/mL FITC-P-113 and FITC-P-113Tri for 2 min. The mean fluorescence intensity (MFI) was measured and compared among the cells treated with these two peptides. As shown in Figure 4B, peptide binding to the NGY152 strain was defined as 100%. Mnt1 and Mnt2 are α-1,2-mannosyl transferases that add mannose residues in *O*-glycosylation. The binding of peptides to the *mnt1*-, *mnt2*-, and *mnt1mnt2*-deletion mutants was similar to that of the parental NGY152 strain.

Among the other *C. albicans* enzymes related to *N*-mannosylation, Mnt4 and Mnt5 participated in *N*-mannan branching [31]. The binding of P-113 and P-113Tri to the *mnt4mnt5*-deletion mutants decreased by 56% and 40%, respectively, compared to the parental NGY152 strain. Och1 is responsible for adding the first α-1,6-mannose to the inner core, and inactivation of Och1 blocks the elongation of *N*-mannans [32]. Moreover, Mnn4, Mnt3, and Mnt5 mediate the addition of β-1,2-linked and α-1,6-linked mannose residues during *N*-mannosylation [31,33,34]. Mnt, Mnn, and Och1 all require Mn^++^ for their functions and therefore depend on the Golgi Mn^++^ transporter Pmr1. The *pmr1*Δ (NGY355) mutant was defective in *N*-linked and *O*-glycosylation [35]. The overall peptide binding to the *mnt3mnt5*-, *pmr1*-, *och1*-, and *mnn4*-deletion mutants were largely decreased compared to the parental NGY152 strain (Figure 4B).

The binding of the FITC-conjugated peptide to the mannosylation-defective mutants was also examined using confocal microscopy (Figure 4C). We found that P-113 and P-113Tri can bind to NGY337 (*mnt1*Δ*mnt2*Δ), NGY516 (*mnt4*Δ*mnt5*Δ), and NGY1227 (*mnt3*Δ*mnt5*Δ). Notably, binding of the peptides to CDH15 (*mnn4*Δ) and NGY357 (*och1*Δ) mutants was almost undetectable. Together, these results suggest that peptides bind to *N*-linked mannan but not *O*-linked mannan.

To further reveal the relationship between the activity of the peptides and protein mannosylation, minimum inhibitory concentrations (MICs) of the peptides were determined using *C*. *albicans* mannosylation-defective mutants (Table 2). For *O*-mannosylation, mutant strains of the protein mannosyltransferase (PMT) family, Mnt1 and Mnt2, were tested. The MICs of P-113 and P-113Tri were 6 and 1.5 μg/mL, respectively, for the CAF2-1 strain. In addition, the MIC values of the *pmt2*-, *pmt4*-, and *pmt6*-deletion mutants were similar to that of the parental CAF2-1 strain. Notably, the *pmt5*-deletion mutant was more resistant to P-113 (MIC = 12 μg/mL) than the CAF2-1 strain. Pmt1, Pmt2, Pmt4, Pmt5, and Pmt6 are members of the PMT family that are required for the initiation of *O*-glycosylation. The MICs of P-113 and P-113Tri for the control NGY152 strain were 12 and 3 μg/mL, respectively. The *mnt1*-deletion mutant was slightly more sensitive to P-113 and P-113Tri compared to its parental NGY152 strain. However, the *mnt2*-deletion mutant was more resistant to P-113, and the *mnt1*Δ/*mnt2*Δ double mutant was more resistant to P-113Tri compared to the NGY152 strain (Table 2).

For *N*-mannosylation, mutant strains of Mnt3, Mnt4, Mnt5, Och1, and Mnn4 were tested. In Table 2, the *mnt3*Δ*mnt5*Δ (NGY1227), *mnt4*Δ*mnt5*Δ (NGY516), *och1*Δ (NGY357), and *mnn*4Δ (CDH15) mutants were more resistant to P-113 (MIC ≥ 24 μg/mL) compared to their parental NGY152 strain (MIC = 12 μg/mL). Finally, both *och1*Δ and *mnn4*Δ mutants were resistant to P-113Tri. Moreover, the *pmr1*Δ (NGY355) mutant was more sensitive to P-113 than the NGY152 strain (Table 2). Together, the peptides binding to mannan, particularly *N*-linked mannan, have a partial effect on their activity against *C*. *albicans*.

### 3.4. Binding to Phosphomannan Also Plays a Profound Role in The Activity of The Peptides

The *N*-linked mannan contains an α-1,6-linked polymannose backbone attached with side chains (consisting of α-1,2- and α-1,3-linked oligomannosides and β-1,2-linked mannose residues), and a mannosylphosphate-containing fraction is attached to the side chains via phosphodiester bonds [36]. Moreover, approximately 20% of the cell wall of phosphomannan has also been found to attach to the *O*-linked mannan [37]. Interestingly, several studies indicate that the binding of antifungal proteins and synthetic peptides is reduced to glycosylation mutants with the loss of phosphomannan and concomitant reduction in cell surface negative charge [36,38,39]. The reduced binding in turn enhances cellular resistance to antifungal proteins and peptides, suggesting that these antifungal agents can recognize the cell surface patterns of the pathogens to maximize their efficacy. Therefore, it is interesting to determine the possible binding of our peptides to phosphomannan and the impact of this binding on the anti-*Candida* activity of the tested peptides.

In Figure 4B, the ratio of P-113 binding to various mutants is presented with shades of blue. The deep blue represents the strongest P-113 binding to the mutant strains, and the white represents the weakest binding of P-113. Figure 4B shows that the binding of P-113 and P-113Tri to *pmr1*Δ, *mnt3*Δ*mnt5*Δ, *mnn4*Δ, and *och1*Δ were largely decreased compared to the parental NGY152 strain. Importantly, all of these mutant strains also play an important role in phosphomannan biosynthesis. Therefore, these data in Figure 4B suggest that P-113 and P-113Tri can also bind to phosphomannan.

To further determine the possible differences of P-113 and P-113Tri binding to negatively charged phosphate, we tested the effect of exogenous glucosamine 6-phosphate, monosaccharide mannose 6-phosphate-BSA, trisaccharide mannose 6-phosphate-BSA, and pentasaccharide mannose 6-phosphate-BSA on the antifungal efficacy of P-113 and P-113Tri. As shown in the MIC assay (Table 3), 5 or 10 mM glucosamine 6-phosphate had no significant adverse effects on the efficacy of P-113Tri to inhibit *C*. *albicans* cell growth compared to the controls without glucosamine 6-phosphate. However, glucosamine 6-phosphate significantly reversed the efficacy of P-113 to inhibit *C*. *albicans* (Table 3). Interestingly, 5 mg/mL monosaccharide mannose 6-phosphate-BSA, trisaccharide mannose 6-phosphate-BSA, and pentasaccharide mannose 6-phosphate-BSA significantly reversed the inhibition of *C. albicans* growth caused by P-113 and P-113Tri. These results indicated that the negative charge on the glycan also contributes to the efficacy of the peptides against *C*. *albicans*.

### 3.5. Screening the Potential Glycan Targets of P-113Tri by Glycan Array

**Figure 5:**
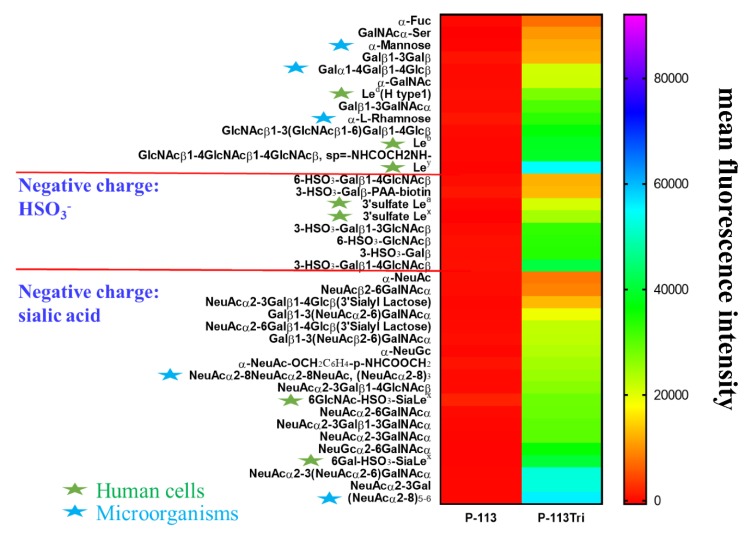
Screening of carbohydrate targets for the peptides using a solution carbohydrate array. The array contained different terminal sequences of *N*-glycans, *O*-glycans, and glycosphingolipids of microbial cells and mammalian tissues. Briefly, donor beads (500 ng/well) and biotin-PAA-sugars were mixed with FITC-P-113 and FITC-P-113Tri. Subsequently, a mixture of acceptor beads, mouse anti-FITC antibody, and rabbit anti-mouse IgG antibody was added. The binding signals were analyzed with a PerkinElmer Envision instrument using AlphaScreen^TM^. The top 40 glycans with peptide binding are shown. Le^d^ (H type 1): Fucα1-2Galβ1-3GlcNAcβ, Le^b^: Fucα1-2Galβ1-3(Fucα1-4)GlcNAcβ, Le^y^: Fucα1-2Galβ1-4(Fucα1-3)GlcNAcβ, 3’sulfate Le^a^: 3-HSO_3_-Galβ1-3(Fucα1-4)GlcNAcβ, 3’sulfate Le^x^: 3-HSO_3_-Galβ1-4(Fucα1-3)GlcNAcβ, 6GlcNAc-HSO_3_-Sia Le^x^: Neu5Acα2-3Galβ1-4(Fucα1-3)(6-HSO_3_)GlcNAcβ, 6Gal-HSO_3_-Sia Le^x^: Neu5Acα2-3(6-HSO_3_)Galβ1-4(Fucα1-3)GlcNAcβ. Gal: galactose; GalNAc: N-acetylgalactosamine; Glc: glucose; GlcNAc: N-acetylglucosamine; NeuAc: N-acetylneuraminicacid; NeuGc: N-glycolylneuraminicacid. Green stars: glycans presented in human cells; Blue stars: glycans presented in microbial cells.

To further understand the carbohydrate/P-113Tri interaction, we determined whether P-113Tri can recognize and bind to specific glycans that commonly do not exist in *C*. *albicans* using a homogeneous solution carbohydrate array [25]. In this array, there were 106 synthetic glycans that represent the terminal sequences found on *N*-glycans, *O*-glycans, and glycosphingolipids of different microbial cells and mammalian tissues. Figure 5 shows that the 40 most common glycans were bound by P-113Tri. For example, P-113Tri bound to α-mannose monohydrate with a fluorescence intensity of ~15,000 (Figure 5). This result further verified that P-113Tri can recognize and interact with the α-mannose moiety on the *C. albicans* cell surface. In addition, P-113Tri can strongly interact with the glycans designed as Le^d^ (H type 1), Le^b^, Le^y^, and 6GlcNAc-HSO_3_-SiaLe^x^, and 6Gal-HSO_3_-SiaLe^x^, which are generally found to localize in the terminal structure of *N*-linked glycans of mammalian tissues and are commonly used as cancer markers. In addition, P-113Tri bound to the glycans α-L-rhamnose, Galα1-4Galβ1-4Glcβ, (NeuAcα2-8)_3_, and (NeuAcα2-8)_5−6_, which are found to generally localize on the bacterial cell surface. Interestingly, P-113 did not bind to any glycans we tested (Figure 5). The results suggest the differences in P-113Tri and P-113 binding glycans with different specificities.

## 4. Discussion

In this study, we investigated the difference between P-113Tri and P-113 in their anti-*C*. *albicans* activity. P-113 was found to translocate rapidly through the cell surface and accumulate intracellularly. However, although small amounts of P-113Tri slowly gained access to the cells, most of the P-113Tri remained associated with the *C*. *albicans* cell surface (Figure 1A–D), particularly the carbohydrates of the cell wall (Figure 2A−E and Figure 3B). Importantly, this carbohydrate/peptide interaction is related to the candidacidal activity of P-113Tri (Figure 2F). The impacts of microbial cell wall components on the activity of cationic AMPs have been reported in several studies [40,41,42,43]. For example, *Staphylococcus aureus* mutants defective in teichoic acids, a major cell wall component, have increased sensitivity to defensins, protegrins, and other AMPs [42]. Human β-defensin-3 and α-defensin-1 exert antibacterial activity by binding to lipid II of Gram-positive bacteria [44,45]. Moreover, aculeacin A and nikkomycin Z inbibit fungal 1,3-β-glucan synthase and chitin synthases, respectively [46,47]. Moreover, Hst 5 binding to the β-glucans of the cell wall is required for candidacidal activity [48]. A previous study also showed that a specific sequences of P-113 can be recognized by transporter (i.e., independent of cell wall binding) for intracellular translocation [17]. In the present study, after cell incubation with peptides for ~5 min, most P-113 readily gained access into the cells (Figure 1) and this result is somehow consistent with Jang et al. [17]. Interestingly, P-113Tri largely remained on the cell surface even after incubation for ~5 min and ~ 1 h (Figure 1). One possible explanation for these results is that the high net charge and alpha-helical content of P-113Tri [19] may contribute to the enhanced interaction between P-113Tri and cell surface, leading to retain on the cell wall. Another possible explanation is that the conformation changes of P-113Tri (compared to P-113) somehow interfere the peptide–cell surface interaction. Moreover, we are not sure whether transporter(s) are involved in the transportation of P-113Tri. Further study is needed to address these questions.

In this work, Zymolyase and sodium metaperiodate were used to remove β-1,3-glucan and β-1,6-glucan from the cell wall, respectively. The cells treated with Zymolyase showed a significant reduction in their interaction with the peptides compared to the cells treated with metaperiodate (Figure 2C and 2D). In addition, cells preincubated with laminarin (mainly consisting of β-1,3-glucan) can decrease the binding of the peptides to *C*. *albicans* cells (Figure 3A). Moreover, cells treated with Zymolyase showed lower sensitivity to the peptides compared to the cells without peptide treatment (Figure 2F), and adding laminarin can rescue the cells from the candidacidal effect of the peptides (Figure 3B). Therefore, our results suggest that the binding of peptides to the β-1,3-glucan layer plays an important role in the activity of AMPs. These results are consistent with Han et al. [23], who demonstrated that the antimicrobial activities of Hst 5 and P-113 were drastically decreased in cells treated with laminarin but not pustulan (mainly consisting of β-1,6-glucan). Interestingly, the binding of P-113Tri to ConA-treated cells also decreased (Figure 2A). This result is in agreement with the glycan array screening (Figure 5), in which P-113Tri bound to α-mannose monohydrate (Figure 5). Moreover, the binding of P-113 and P-113Tri was reduced in cells treated with α1-2,3,6 mannosidase, which can remove the mannan layer of the cell wall (Figure 2B), and the competition assay using mannan also showed a decrease in the activity of the peptides (Figure 3A). Therefore, our results further indicate that the mannan layer plays an important role in mediating the function of the peptides.

To further investigate the influence of mannan on the activity of peptides, *C*. *albicans* mutant strains defective in *O*-linked and *N*-linked mannan were used. We found that *N*-linked mannan and phosphomannan, but not *O*-linked mannan, on the cell surface can affect P-113Tri binding (Figure 4B,C). However, the candidacidal activity of P-113Tri is not correlated with the binding ability of P-113Tri to *N*-linked mannan and phosphomannan. In Table 2, *och1*Δ (NGY357) and *mnn*4Δ (CDH15) mutants were more resistant to P-113Tri compared to the control NGY152 strain, which were correlated to the binding ratio of P-113Tri. However, the *mnt3*Δ*mnt5*Δ (NGY1227), *mnt4*Δ*mnt5*Δ (NGY516), and *pmr1*Δ (NGY355) mutants were more sensitive or similar to P-113Tri compared to the control NGY152 strain. These data suggest that the binding of P-113Tri to the *N*-linked mannan and phosphomannan on the *Candida* surface is not the only mechanism for the killing ability of P-113Tri. Because the cell wall is the outmost layer of *C. albicans* cells, we only focused on the cell wall–peptide interaction in this study and found that specific cell wall glycans are related to the candidacidal activity of P-113 and P-113Tri. In our previous study [49], we found that the peptides can somehow also target to the mitochondria. However, the mechanism through which the peptides cross the cell surface and affect the mitochondrial functions is still under investigation. It is worth noting that only cells that have totally lost phosphomannan, mannan, and glucan (*och1*Δ and *mnn*4Δ mutants, and Zymolyase-treated cells) showed the reduced candidacidal activity from P-113Tri. Moreover, previous studies showed that physiological conditions, such as blood or serum, decreased the structural complexity of mannan in the *C*. *albicans* cell wall [50]. In addition, *Candida* clinical isolates also showed increased glucan exposure through the loss of the acid-labile mannan structure [51]. Therefore, P-113Tri can specifically and strongly bind to *N*-linked mannan and phosphomannan and can have potent antifungal activity against *C. albicans* with a reduced mannan content. Together, these data suggest that P-113Tri can be a potential antifungal drug in the future. In a previous study, the loss of negatively charged *N*-linked phosphomannans from the cell wall enhanced *C. albicans* resistance to the AMP dermaseptin by reducing peptide binding and entry from the cell surface [36]. In addition, exogenous phosphosugars were able to reduce the efficacy of P-113 and P-113Tri (Table 3). Moreover, it seems that the binding of cationic peptides to the *C. albicans* cell wall is not only related to the net charge of the cell surface but may also involve pattern recognition of *C*. *albicans* by the AMPs. This possibility requires further investigation.

Using a glycan array, P-113Tri can bind to carbohydrates commonly found on the bacterial cell wall and on human cells, including some cancer glycan epitopes (Figure 5). For example, P-113Tri has a strong binding to Le^y^, Le^b^, Le^a^, Le^x^, and sLe^x^ [48]. The Le^y^ epitope is found in lung, urinary bladder, prostate, ovarian, head, and neck and thymus cancer cells, whereas the Le^b^ epitope exists in urinary bladder, and endometrial cancer cells. Moreover, the Le^a^ epitope is present in stomach, pancreatic, lung, and endometrial cancer cells, whereas the Le^x^ is found in pancreatic, lung, kidney, urinary bladder, breast, head, and neck cancer cells. Finally, sLe^x^ has been identified in stomach, pancreatic, lung, prostate, and breast cancer cells [52]. Interestingly, the binding of P-113 to all the glycans in the tested array was not detectable (Figure 5). These results suggest that P-113Tri and its parental P-113 have a fundamental difference in their carbohydrate recognition and binding, and P-113Tri can possibly bind to different cancer cells to facilitate its anticancer activity. Further study is required to determine the potential of P-113Tri as an anticancer agent.

Although AMPs bind to carbohydrates, we cannot exclude that the peptides may also interact with cell wall proteins that consist of 20%–30% (in mass) of the fungal cell wall [27]. In particular, mannan is covalently bound to proteins (mannoproteins), and this form of mannan accounts for 40% of the total cell wall polysaccharides on the *C. albicans* exterior [53]. Mannoproteins are involved in cell–cell recognition and trigger immune responses [54]. Several studies have shown that AMPs function via their interactions with microbial cell wall proteins. For example, *C*. *albicans* Ssa1/2 proteins are required for Hst 5 binding [55]. The *C. albicans* proteins possibly targeted by P-113Tri and the significance of such AMP/protein interactions are currently under investigation. Moreover, in our previous study [19], P-113Tri showed an overall higher efficiency to kill various *C. albicans* and non-albicans *Candida* clinical isolates than P-113. However, all the *C. tropicalis* reference strain ATCC 13803 and three clinical isolates (YH50007, YH50013 and YH50114) were sensitive to both P-113 and P-113Tri (Table 2 of [19]). Recently, Navarro-Arias et al. compared the content of chitin, mannan, glucan, and phosphomannan on *C. tropicalis* and *C. albicans* cell wall and found that there is no significant difference in these two species [56]. However, the porosity of *C. tropicalis* cell wall is higher than *C. albicans* (63% vs. 28%) [56], which raises a possibility that P-113 is much easier to gain access into the *C. tropicalis* cells than P-113Tri due to the small size of P-113 and has a higher activity against *C. tropicalis* than *C. albicans*. Further study is needed to test this possibility.

Many studies have revealed that peptides with higher net charge and alpha-helical content are generally prone to have strong antimicrobial activity [43]. For example, Ma et al. showed that a peptide with a tandem arrangement of two leucine-rich repeats (LRRs) forms alpha-helical structure and possesses a higher antibacterial activity than that containing only one LRR with a random coil structure [57]. Moreover, this study also suggests that the secondary structure plays a more vital role in antimicrobial activity of peptides than the primary sequence of peptides. For the peptides that we studied, using circular dichroism (CD), and the beta-structure selection method, the alpha-helical content of P-113Tri and P-113 is 21.4% and 2.9%, respectively [19]. In addition, the net charges of P-113Tri and P-113 are +15 and +5, respectively. Therefore, other than the secondary structure, the higher net charge of P-113Tri may also contribute to the higher activity of P-113Tri compared to P-113. The relationship between the net charge and secondary structure of these peptides to the selective glycan binding is required to be further analyzed using a molecular dynamic (MD) simulation and/or nuclear magnetic resonance (NMR) spectroscopy.

In addition, *C. albicans* is recognized by the pattern-recognition receptors (PRRs) of host monocytes, macrophages, dendritic cells, and neutrophils through their interaction with different pathogen-associated molecular patterns (PAMPs) on the fungal cell wall. These PAMPs include *N*-linked mannans, *O*-linked mannans, phosphomannan, and β-glucans [58]. The PRR/PAMP interaction induces pro- and anti-inflammatory cytokines, phagocytosis-mediated fungal killing, and represents a crucial mechanism that allows the innate immune system to combat *Candida* infections. Interestingly, neutrophils can secrete a range of AMPs, such as LL-37 and HNP 1-3, to kill microorganisms [59]. Intriguingly, our results imply that not only have immune cells evolved to recognize cell-surface molecular patterns of the fungal pathogens but also AMPs can recognize the same or similar molecular patterns to maximize their efficacy and specificity for pathogen killing.

In summary, the cell wall is the first contact point between fungal pathogens and their environments. Therefore, the composition and structure of the fungal cell wall are important for immune recognition. AMPs are key components of the innate host defense, and the interaction between AMPs and fungal cells is complex because of their association with different components of the cell wall. Our findings here highlight that the tested peptides, particularly P-113Tri, bind to specific glycans (*N*-linked mannan and phosphomannan of the *C. albicans* cell wall) and suggest that these peptides can be modified in future motifs to enhance the interaction between the peptide and the microbial cell surface for potential antifungal therapy.

## Figures and Tables

**Table 1:** *C. albicans* deletion mutants that were P-113 resistant.

No	Systematic Name	Gene Name	Description
**1**	orf19.7590		Putative NADH-ubiquinone oxidoreductase
**2**	orf19.7247	*RIM101*	Transcription factor
**3**	orf19.7391	*OCH1*	α-1,6-mannosyltransferase
**4**	orf19.13191	*SNF4*	Putative subunit of the AMP-activated Snf1p kinase
**5**	orf19.287	*NUO2*	NADH-ubiquinone oxidoreductase subunit
**6**	orf19.1625		Putative ubiquinone oxidoreductase
**7**	orf19.1710	*ALI1*	Putative NADH-ubiquinone oxidoreductase
**8**	orf19.2570	*MCI4*	Putative NADH-ubiquinone dehydrogenase
**9**	orf19.2821		Protein of unknown function
**10**	orf19.4758		Putative reductase or dehydrogenase
**11**	orf19.5547		Protein of unknown function
**12**	orf19.3995	*RIM13*	Protease of the pH-response pathway
**13**	orf19.4755	*KEX2*	Subtilisin-like protease
**14**	orf19.5068	*IRE1*	Putative protein kinase
**15**	orf19.6293	*EMP24*	COPII-coated vesicle component

**Table 2:** The minimum inhibitory concentrations (MICs) of P-113 and P-113Tri against *C. albicans* glycosylation mutants.

	MIC_90_ (μg/mL) ^a^ in LYM broth	
	P-113	P-113Tri
***O*-linked Glycosylation Mutants**		
CAF2-1 (Control)	6	1.5
SPCa2 (Pmt1)	ND ^b^	ND ^b^
SPCa4 (Pmt2)	6	1.5
SPCa6 (Pmt4)	6	3
SPCa8 (Pmt6)	6	3
SPCa10 (Pmt5)	12	3
NGY152 (Control)	12	3
NGY145 (Mnt2)	24	3
NGY158 (Mnt1)	6	1.5
NGY337 (Mnt1Mnt2)	12	6
***N*-linked glycosylation mutants**		
NGY152 (Control)	12	3
NGY516 (Mnt4Mnt5)	24	1.5
NGY1227 (Mnt3Mnt5)	>24	1.5
NGY357 (Och1)	>24	12
CDH15 (Mnn4)	24	>12
***O* and *N*-linked glycosylation mutants**		
NGY355 (Pmr1)	3	3

^a^ The MIC_90_ was measured after the cells were incubated with each peptide for 48 h. ^b^ ND: Not determined.

**Table 3:** Exogenous phosphosugars reduce the anti-*C. albicans* activity of the peptides.

	MIC_90_ (μg/mL) ^a^ in LYM broth	
	P-113	P-113Tri
Control	3	0.75
5 mM Glucosamine 6-phosphate	6	1.5
10 mM Glucosamine 6-phosphate	12	1.5
5 mg/mL monosaccharide mannose 6-phosphate-BSA	>24	12
5 mg/mL trisaccharide mannose 6-phosphate-BSA	24	6
5 mg/mL pentasaccharide mannose 6-phosphate-BSA	>24	6

^a^ The MIC_90_ was measured after the cells were incubated with each peptide for 48 h.

## Supplementary Materials

The following are available online at https://www.mdpi.com/2076-2607/8/2/299/s1, Figure S1. 3D structure of P-113 and P-113Tri predicted by I-TASSER (iterative threading assembly refinement). Figure S2. Aniline blue binding to zymolyase-treated *C. albicans*. Table S1. *C. albicans* strains used in this study.
