# Supplementary material for: The interaction between Carbohydrates and the Antimicrobial Peptide P-113Tri is Involved in the Killing of Candida albicans
Source: Microorganisms. 2020 Feb 21;8(2):299. doi: 10.3390/microorganisms8020299 (PMC7074873; doi:10.3390/microorganisms8020299)
Supplement: Supplementary file 1 [file microorganisms-08-00299-s001.pdf]

**Table S1. *C. albicans* strains used in this study.**

| Strain | Genotype | Source |
|---|---|---|
| ***C. albicans*** | | |
| SC5314 | Wild type | [1] |
| CAF2-1 | *ura3Δ::imm434/URA3* | [2] |
| SPCa2 | *pmt1Δ::hisG/pmt1Δ::hisG ura3Δ::imm434/URA3* | [3] |
| SPCa4 | *pmt2Δ::hisG/PMT2 ura3Δ::imm434/URA3* | [3] |
| SPCa6 | *pmt4Δ::hisG/pmt4Δ::hisG ura3Δ::imm434/URA3* | [3] |
| SPCa8 | *pmt6Δ::hisG/pmt6Δ::hisG ura3Δ::imm434/URA3* | [3] |
| SPCa10 | *pmt5Δ::hisG/pmt5Δ::hisG ura3Δ::imm434/URA3* | [3] |
| NGY152 | *ura3Δ: : imm434/ura3Δ: : imm434, RPS1/rps1Δ : :* CIp10 | [4] |
| NGY145 | *ura3Δ : : imm434/ura3Δ : : imm434, mnt2Δ : : hisG/mnt2Δ : : hisG, RPS1/rps1Δ : :* CIp10 | [5] |
| NGY158 | *ura3Δ : : imm434/ura3Δ : : imm434, mnt1Δ : : hisG/mnt1Δ : : hisG, RPS1/rps1Δ : :* CIp10 | [5] |
| NGY337 | *ura3Δ : : imm434/ura3Δ : : imm434, mnt1-mnt2Δ : : hisG/mnt1-mnt2Δ : : hisG, RPS1/rps1Δ : :* CIp10 | [5] |
| NGY516 | ura3Δ: : imm434/ura3Δ: : imm434, *mnt4::hisG/mnt4Δ::hisG, mnt5Δ::dp1200/mnt5Δ::dp1200, RPS1/rps1Δ::*CIp10 | [6] |
| NGY1227 | *ura3Δ : : imm434/ura3Δ : : imm434, mnt3Δ : : dp1200/mnt3Δ : : dp1200, mnt5Δ : : hisG/mnt5Δ : : hisG, RPS1/rps1Δ : :* CIp10 | [6] |
| NGY357 | *ura3Δ : : imm434/ura3Δ : : imm434, och1Δ : : hisG/ och1Δ : : hisG, RPS1/rps1Δ : :* CIp10 | [7] |
| CDH15 | *ura3Δ : : imm434/ura3Δ : : imm434, mnn4Δ : : hisG/mnn4Δ : : hisG, RPS1/rps1Δ : :* CIp10 | [8] |
| NGY355 | *ura3Δ: : imm434/ura3Δ : : imm434, pmr1Δ : : hisG/ pmr1Δ : : hisG, RPS1/rps1Δ : :* CIp10 | [9] |
| SN250 | Wild type | [10] |
| *och1* | From Noble's mutant library | [10] |


1. Gillum, A. M.; Tsay, E. Y.; and Kirsch, D. R. Isolation of the *Candida albicans* gene for orotidine-5'-phosphate decarboxylase by complementation of *S. cerevisiae ura3* and *E. colipyrF* mutations. *Molecular & General Genetics.* **1984,** 198, 179-182.
2. Fonzi, W. A.; and Irwin, M. Y. Isogenic strain construction and gene mapping in *Candida albicans*. *Genetics.* **1993**, 134, 717-728.
3. Prill, S. K.; Klinkert, B.; Timpel, C.; Gale, C. A.; Schroppel, K.; and Ernst, J. F. PMT family of *Candida albicans*: five protein mannosyltransferase isoforms

affect growth, morphogenesis and antifungal resistance. *Mol Microbiol.* **2005**, 55, 546-560.

4. Brand, A.; MacCallum, D. M.; Brown, A. J.; Gow, N. A.; and Odds, F. C. Ectopic expression of URA3 can influence the virulence phenotypes and proteome of *Candida albicans* but can be overcome by targeted reintegration of URA3 at the RPS10 locus. *Eukaryot Cell.* **2004**, 3, 900-909.
5. Munro, C.A.; Bates, S.; Buurman, E.T.; Hughes, H.B.; Maccallum, D.M.; Bertram, G.; Atrih, A.; Ferguson, M.A.; Bain, J.M.; Brand, A., et al. Mnt1p and Mnt2p of *Candida albicans* are partially redundant alpha-1,2-mannosyltransferases that participate in O-linked mannosylation and are required for adhesion and virulence. *J. Biol Chem.* **2005**, *280*, 1051–1060, doi:10.1074/jbc.M411413200.
6. Mora-Montes, H.M.; Bates, S.; Netea, M.G.; Castillo, L.; Brand, A.; Buurman, E.T.; Diaz-Jimenez, D.F.; Jan Kullberg, B.; Brown, A.J.; Odds, F.C.; et al. A multifunctional mannosyltransferase family in *Candida albicans* determines cell wall mannan structure and host-fungus interactions. *J Biol Chem.* **2010**, *285*, 12087–12095, doi:10.1074/jbc.M109.081513.
7. Bates, S.; Hughes, H.B.; Munro, C.A.; Thomas, W.P.; MacCallum, D.M.; Bertram, G.; Atrih, A.; Ferguson, M.A.; Brown, A.J.; Odds, F.C., et al. Outer chain N-glycans are required for cell wall integrity and virulence of *Candida albicans*. *J. Biol. Chem.* **2006**, *281*, 90–98, doi:10.1074/jbc.M510360200.
8. Hobson, R.P.; Munro, C.A.; Bates, S.; MacCallum, D.M.; Cutler, J.E.; Heinsbroek, S.E.; Brown, G.D.; Odds, F.C.; Gow, N.A. Loss of cell wall mannosylphosphate in *Candida albicans* does not influence macrophage recognition. *J. Biol. Chem.* **2004**, *279*, 39628–39635, doi:10.1074/jbc.M405003200.
9. Bates, S.; MacCallum, D.M.; Bertram, G.; Munro, C.A.; Hughes, H.B.; Buurman, E.T.; Brown, A.J.; Odds, F.C.; Gow, N.A. *Candida albicans* Pmr1p, a secretory pathway P-type $Ca^{2+}/Mn^{2+}$-ATPase, is required for glycosylation and virulence. *J. Biol. Chem.* **2005**, *280*, 23408–23415, doi:10.1074/jbc.M502162200.
10. Noble, S.M.; French, S.; Kohn, L.A.; Chen, V.; Johnson, A.D. Systematic screens of a *Candida albicans* homozygous deletion library decouple morphogenetic switching and pathogenicity. *Nat. Genet.* **2010**, *42*, 590–598, doi:10.1038/ng.605.

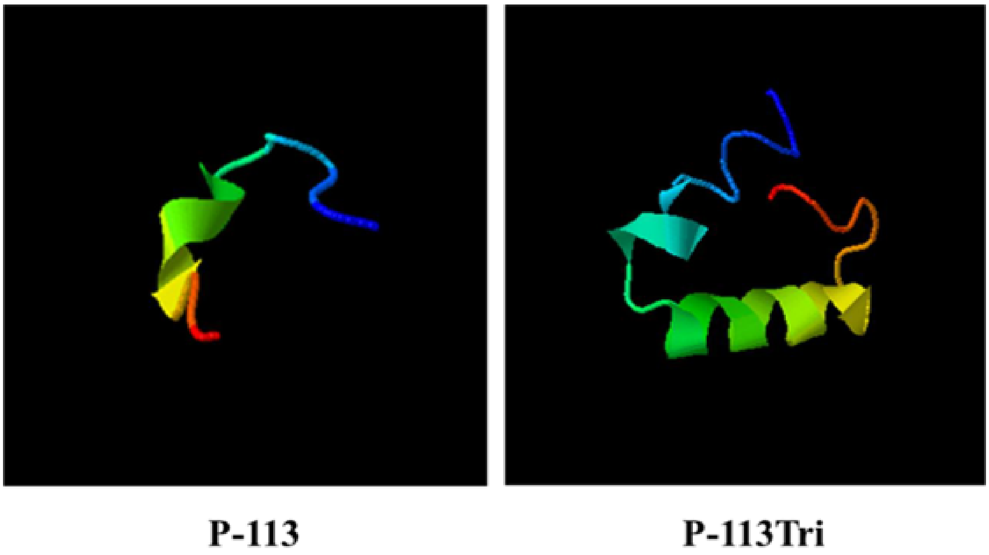


**Figure S1. 3D structure of P -113 and P-113Tri predicted by I-TASSER (Iterative threading assembly refinement).**

P-113 shows a coil structure, whereas P-113Tri exhibits an α-helical structure. I-TASSER on-line platform (https://zhanglab.ccmb.med.umich.edu/I-TASSER/).

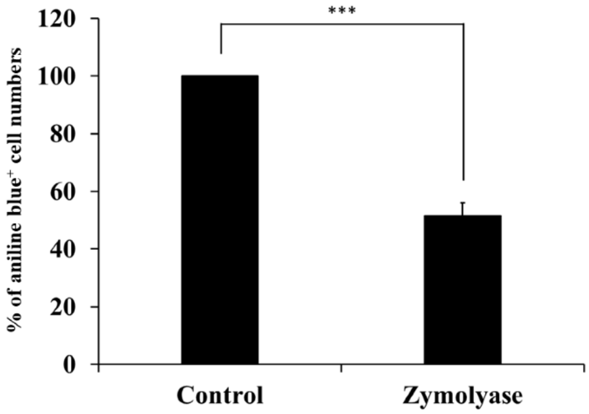


**Figure S2. Aniline blue binding to zymolyase-treated *C. albicans*.**

*C. albicans* cells were treated with Zymolyase (2.5 mg/ml) for 1 h at 37℃. Then, the cells were treated with aniline blue (500 μg/ml) for 5 min in a black 96-well microplate. *, $p<0.05$; **, $p<0.01$; ***, $p<0.001$.
